# Validating performance status and activities of daily living assessment tools for Chinese palliative care in a cancer setting: A cross-cultural psychometric study

**DOI:** 10.1016/j.apjon.2024.100613

**Published:** 2024-10-29

**Authors:** Yunyun Dai, Jinfeng Ding, Barbara A. Daveson, Yongyi Chen, Alanna Connolly, Claire E. Johnson

**Affiliations:** a Hunan Cancer Hospital, The Affiliated Cancer Hospital of Xiangya School of Medicine, Central South University, Changsha, China; b Faculty of Science, Medicine and Health, University of Wollongong, Wollongong, Australia; c School of Nursing, Guilin Medical University, Guilin, China; d Xiangya School of Nursing, Central South University, Changsha, China; e Palliative Care Outcomes Collaboration (PCOC), Faculty of Science, Medicine and Health, University of Wollongong, Wollongong, Australia; f Palliative Aged Care Outcomes Program (PACOP), Faculty of Science, Medicine and Health, University of Wollongong, Wollongong, Australia

**Keywords:** Palliative care, Point of care resources assessment, Australia-Modified Karnofsky Performance Status, Resource Utilization Groups-Activities of Daily Living, Validity, Reliability

## Abstract

**Objective:**

National approaches to the routine assessment of palliative care patients improve patient outcomes. However, validated tools and a national methodology for this are lacking in Mainland China. The Australian Palliative Care Outcomes Collaboration (PCOC) model is a well-established national program aimed at improving the quality of palliative care based on point-of-care outcomes assessment. This study aimed to culturally adapt and validate two measures used in PCOC (Australia-modified Karnofsky Performance Status [AKPS], Resource Utilization Groups - Activities of Daily Living [RUG-ADL]) in the Chinese context.

**Methods:**

A cross-cultural adaptation and validation study involving forward and backward translation methods, cognitive interviewing, and psychometric testing.

**Results:**

Two minor adjustments were made to the scoring instructions for the RUG-ADL, and the AKPS remained unchanged. Twenty-two clinicians participated in psychometric testing, completing 363 paired assessments on 135 inpatients. The correlations between AKPS and the Barthel index (BI) for activities of daily living (*r* = 0.77, *P* < 0.001), AKPS and RUG-ADL (*r* = −0.82, *P* < 0.001), RUG-ADL and BI (*r* = −0.67 to −0.76) demonstrated good concurrent validity for both the AKPS and the RUG-ADL. The inter-rater reliability for AKPS (*k* = 0.63) and RUG-ADL were substantial and moderate (*k* = 0.51–0.56), respectively. The RUG-ADL also showed good internal consistency (Cronbach's alpha = 0.92). Both tools were able to detect patients' urgent needs.

**Conclusions:**

The Chinese version of AKPS and RUG-ADL can be systematically used to assess performance status and dependency among palliative care patients. However, observational assessments and enhanced communication between clinicians and patients/caregivers is also recommended for optimal clinical utility.

## Introduction

Performance status, activities of daily living (ADL) capacity, and symptom assessments are collectively regarded as the “vital signs” for palliative care patients.^1^ Integrating these assessments into routine clinical practice enables accurate and dynamic reflection of the care needs of patients and their families or caregivers.^1^^,^^2^ This approach assists clinicians in planning and delivering tailored, holistic care based on point-of-care data collection. Moreover, performance status serves as an essential predictive marker for patients’ outcomes, such as prognosis, and aiding in the making of informed decisions that align with the goals of the patient and their family.^3^^,^^4^ Additionally, assessing patients' ADL capacity can inform the level of support families, caregivers or health care professionals need and helps optimize resources allocation.^5^

With Mainland China experiencing an aging population surge coupled with an increasing incidence of cancer and chronic diseases, the demand for palliative and end-of-life care is rising.^6^^,^^7^ Since 2017, the Chinese government has launched pilot programs to advance palliative care services, expanding to 185 cities by 2023. Despite these efforts, the development of palliative care in Mainland China is still at a nascent stage. There is a notable deficiency in standardized bedside assessment tools that are tailored to efficiently allocate resources and meet the specific needs of palliative care patients and their families or caregivers.^8^ Moreover, the absence of a nationwide, standardized quality improvement program that emphasizes bedside assessments for palliative care in Mainland China is a pressing concern. The implementation of a well-established, effective and feasible quality improvement program could promote high-quality palliative care, especially during its initial phases of development in Mainland China.

The Palliative Care Outcomes Collaboration (PCOC), which is a national palliative care quality improvement program funded by the Australian Government Department of Health and Aged Care, exemplifies successful integration of palliative care patients' performance status, ADL capacity, symptom assessments and a response framework into routine clinical practice.^9^ A central component of the PCOC model involves routinely assessing the palliative care needs of patients and families/caregivers using five standardized and validated tools (Supplementary Fig. 1):^9^ the Symptom Assessment Scale (PCOC-SAS) for symptom distress,^10^ the Palliative Care Problem Severity Score (PCPSS) for symptoms severity,^11^ the Palliative Care Phase for clinical acuity and urgency,^12^ the Australia-modified Karnofsky Performance Status (AKPS) for performance status^4^ and Resource Utilization Groups - Activities of Daily Living (RUG-ADL) for functional dependency.^13^ Another core component of the PCOC program is the bi-annual benchmark report provided to participating palliative care services. The reports provide consolidated information based on patients' clinical outcomes compared with the national quality benchmarks and identify areas for improvement (Supplementary Fig. 2).^9^ The services participating in PCOC are supported by improvement facilitators who provide education, quality improvement support. Additionally, benchmarking workshops and communities of practice are facilitated to further support these goals.^9^ Within Australia, data have demonstrated that PCOC has led to statistically and clinically significant improvements in patient and carer outcomes over time through routine data collection and systematic feedback of information on performance to palliative care services.^2^^,^^14^ Compared to other quality improvement programs, the PCOC model has several distinct advantages. It facilitates standard assessment and communication among clinicians, provides baseline assessments and snapshots of patient needs, tracks and responds to patients’ symptoms and problems, involves patients, families, and carers in the decision-making process, and drives quality improvement by using evidence-based data.

Considering the gap in the quality of palliative care in Mainland China, our team is pilot testing the PCOC model in a Chinese cancer hospital. We chose a cancer hospital for this pilot because cancer patients constitute the majority of palliative care patients, and many palliative care units in Mainland China are either established within or embedded in cancer departments.^15^^,^^16^ Before formally integrating the PCOC tools and response framework into routine palliative care clinical practice, validating the five PCOC assessment tools in the Chinese context is essential. This study aims to cross-culturally adapt and validate the AKPS and RUG-ADL to support the PCOC model's application in routine clinical practice in mainland China. In Mainland China, tools like the Karnofsky Performance Status (KPS) and the Barthel Index (BI) are commonly used to assess performance status and ADL in cancer patients, including those in palliative care.^17^^,^^18^ However, these tools have limitations in addressing the specific needs of palliative care, such as flexible language and focused assessment of functional dependency without guidance on resources allocation. Therefore, validating the AKPS and RUG-ADL is crucial before integrating the PCOC model into routine practice in China, as these tools offer more targeted assessments aligned with the PCOC framework.

We employed the BI for activities of daily living to test the concurrent validity of both the AKPS and RUG-ADL, and the Palliative Care Phase to assess their known-groups validity. We hypothesize that:

• Concurrent validity: The AKPS and RUG-ADL will show high correlations with the BI, indicating they can effectively assess patients' performance status and functional dependency.
• Known-groups validity: The AKPS and RUG-ADL are highly associated with patients' palliative care phases and distinguish different levels of care needs.
• Reliability: Both tools will exhibit good inter-rater reliability, with the RUG-ADL also demonstrating good internal consistency.

## Methods

The use of the PCOC model, including the RUG-ADL, was authorized by the PCOC at the University of Wollongong for use in this study. As confirmed by its developer, Professor Amy P. Abernethy, the AKPS is freely available for research purposes without the need for formal permission. This study is part of a larger validation project for the five PCOC assessment tools. Another sub-study was reported in our previous publication for the PCOC SAS, PCPSS, and Palliative Care Phase.^19^

### Cross-cultural adaptation component

The cross-cultural adaptation component involved forward and backward translation of the tools and cognitive interviews to enhance the clarity and comprehension of the Chinese version of the tools.

#### Phase 1: Translation of the AKPS and RUG-ADL into simplified Chinese

The translation of the AKPS and RUG-ADL into simplified Chinese followed Brislin's forward-and-backward translation model.^20^ Two postgraduate students, one majoring in Nursing and the other in English Education, independently translated the tools into simplified Chinese. After comparing both versions, inconsistencies were resolved through discussion, resulting in a preliminary Chinese version. This version was then back-translated into English by a third translator, a nurse unfamiliar with the original tools. Two Australian PCOC academic staff with clinical expertise reviewed the back-translation against the original tools, identifying any discrepancies. These were resolved collaboratively among the three Chinese translator team and the two PCOC staff. Finally, the Chinese-speaking research team finalized the Chinese version, addressing any ambiguities.

#### Phase 2: Cognitive interviews

Cognitive interviews^21^ were undertaken to culturally adapt the AKPS and RUG-ADAL. Since both tools are clinician-rated, six Chinese palliative care clinicians (three nurses and three doctors) who had completed core education in the PCOC model (i.e., the PCOC fundamentals education sessions) were invited to use the tools to assess palliative care inpatients.^22^ (Supplementary Table 1: Participants characteristics for cognitive interviews). The “think-aloud” technique and “read-aloud” techniques were employed, where participants verbalized their thoughts while using the tools and reading the items aloud.^23^ Verbal probing (asking questions during tool use) was also used to obtain feedback on the tools’ clarity, comprehensibility, and interpretability^23^ (Supplementary Table 2: Cognitive interview guideline). Field notes were taken during the interviews.

### Psychometric testing component

#### Study settings

The psychometric test was conducted in the 20-bed palliative care unit and the 16-bed advanced cancer care unit at a Cancer Hospital in mainland China. These units provide care for patients within advanced care, combining palliative services with active treatment.

#### Participants

We recruited 25 health care professionals – 15 from both the palliative care unit (three doctors, 12 nurses) and 10 from advanced cancer care units (eight nurses, two doctors) – along with patients admitted for symptom management. Participation occurred between February and April 2023.

#### Measures

##### Australia-modified Karnofsky Performance Status scale (AKPS)

The 11-point AKPS is a clinician-rated scale to capture patients’ functional status from three different aspects: activity, work and self-care. The scores range from 0 (deceased) to 100 (fully functioning) and are scored in increments of 10 (i.e., 10, 20, 30 …). Compared with the KPS and the Thorne-modified KPS (TKPS), AKPS is more appropriate for using in different kinds of palliative care settings as its language focus on assessing performance status without restricting it to judgements on intensity of clinical treatment or the availability of resources.^4^

##### Resource utilisation group-activities of daily living (RUG-ADL)

The RUG-ADL was originally developed as part of a Nursing Home case-mix classification in the USA in 1994^13^ and was subsequently adapted by the PCOC team.^9^ The RUG-ADL is a health professional-completed scale which assesses four activities of daily living specifically eating, toileting, bed mobility and transfers. Scores for the eating item range from one (independent or supervision only) to three (extensive assistance/total dependence/tube fed). Scores for the toileting, bed mobility and transfer items range from one (independent or supervision only) to 5 (two or more-person physical assist), without a score of “2”. This is because, for toileting, bed mobility and transfer, the shift from independent/supervision only (score “1”) to limited assistance (score “3”) was found to equate to a three-fold increase in resources compared to the resources required for eating. The total RUG-ADL score ranges from four to 18, with higher scores indicating lower functional ability. A total RUG-ADL score of 4–5 indicates the need for monitoring of patients, a total score of 6–13 suggests that assistance is required, with patients possibly at risk of falls and developing pressure areas, a total score of 14–17 denotes the need for assistance from one person plus equipment, with a greater risk of falls and pressure areas for patients, and a total score of 18 signifies the requirement for two assistants for all care, indicating an even greater risk of pressure areas for patients.^9^

##### Barthel index for activities of daily living (BI)

The BI is a standardized measurement widely used in clinical practice to assess patients' performance in activities of daily living. It includes 10 fundamental items: feeding, bathing, grooming, dressing, bowel control, bladder control, toilet use, transfers (bed to chair and back), mobility on a level surface and stairs.^24^ The Chinese version of BI has a high inter-rater reliability with Intraclass Correlation Coefficient (ICC) of 0.987, and the Cronbach's *α* coefficient was 0.871–0.915.^25^ Higher scores indicate higher functional ability.

##### Palliative care phase

The Palliative Care Phase is a clinician-rated tool that describes the patient's clinical condition and their family/carers' condition, helping guide decisions about the urgency and level of care required. It categorizes each patient's condition into four non-sequential phases (‘stable’, ‘unstable’, ‘deteriorating’ and ‘terminal’) based on comprehensive clinical assessments.^12^ The tool has demonstrated an acceptable level of inter-rater reliability (weighted Kappa = 0.67) and high acceptability were reported among Australian clinicians.^12^ The Chinese version of Palliative Care Phase has also shown its ability to detected patients' urgent needs, and the Inter-rater reliability was reported as fair (*k* = 0.31).^19^

#### Sample size

The sample size was calculated using PASS 15, focusing on the weighted kappa statistic (*k*) to evaluate agreement between clinicians.^26^ This study is part of a larger validation project for the five PCOC assessment tools, and we based our sample size on the Palliative Care Phase tool, as it required the largest number of paired assessments among the PCOC five tools. We expected the weighted kappa coefficient align with an Australian study that reported an agreement of 0.67 (95% CI = 0.61–0.70) among palliative care professionals.^12^ To achieve 80% statistical power with a 95% confidence interval of 0.15, a minimum of 298 paired assessments was required. Accounting for a 20% attrition rate due to patients not being assessed by two palliative care clinicians within 4 hours, we increased the target to 357 paired assessments.

#### Data collection

Two clinicians working on the same shift in the same inpatient palliative care unit at a cancer hospital used the AKPS, RUG-ADL, BI and the Palliative Care Phase to assess the same palliative care inpatient within one four-hour period. The patient's demographic information was collected from the medical records, and the demographic details of the palliative care clinicians were collected after their completing the PCOC assessments. The raters also recorded the time each assessment was conducted.

#### Data analysis and a priori hypotheses

All data analyses were performed using SPSS 25.0.^27^ Demographic information was presented using descriptive statistics. A significant level (*P*-value) was set at less than 0.05. We assessed the internal consistency, inter-rater reliability, and concurrent validity of the AKPS and RUG-ADL using the following methods and a priori hypotheses.

##### AKPS

*Validity:* The concurrent validity of the AKPS was assessed by examining Spearman correlation coefficients (*r*) between the AKPS score and the BI total score, as well as between the AKPS score and the RUG-ADL total score, given that the AKPS evaluates patients’ performance status through their activity, work, and self-care, while the BI and the RUG-ADL assesses the 10 and four most common daily activities, respectively. A patient was considered to have good performance status if they could perform these activities.^28^ Therefore, our hypothesis was that a high correlation exists between the AKPS score and the BI total score, as well as between the AKPS score and the RUG-ADL total score. For interpretation, correlations were categorized as follows: *r* = 0.30–0.49, low correlation, *r* = 0.50–0.69, moderate, *r* = 0.70–0.89 high, and *r* = 0.90–1.00 very high.^29^ The known-groups validity of the AKPS was assessed by examining its ability to predict the palliative care phases for patients. Informed by prior studies, it is evident that patients in the unstable, deteriorating or terminal phases typically require a more urgent and intensive level of care.^10^^,^^19^ Therefore, we hypothesized that patients with good performance status would be more likely to be in a stable phase, and vice versa. The known-groups validity of the AKPS was analysed using Chi–Square Tests.

*Reliability:* For the inter-rater reliability, we utilized the weighted kappa statistic (*k*) to determine the level of agreement between two raters when using the AKPS. We categorized the AKPS into four levels based on their degree of dependency, as a score less than 70 indicates the need for assistance from others, a score of less than 30 indicates complete dependency:^4^ level 1 = self-dependent (AKPS = 70–100); level 2 = assistance required (AKPS = 50–60); level 3 = increasingly limited mobility (AKPS = 30–40); and level 4 = completely bedfast (AKPS = 10–20). *k* = 0.00–0.20 — “slight” agreement, *k* = 0.21–0.40 — “fair”, *k* = 0.41–0.60 — “moderate”, *k* = 0.61–0.80 — “substantial”, and *k* = 0.81–1.00 — “almost perfect” agreement.^30^

##### RUG-ADL

*Validity:* The concurrent validity of the RUG-ADL was assessed by examining Spearman correlation coefficients (*r*) between the RUG-ADL items score and the BI corresponding items score. As both tools assess patients’ daily activities, we hypothesized that there would be high correlations between the RUG-ADL toileting and BI toilet use, RUG-ADL transfer and BI transfers (bed to chair and back), and RUG-ADL eating and BI feeding. The known-groups validity of the RUG-ADL was assessed by examining its ability to predict the palliative care phases for patients. Similarly, with the AKPS, we hypothesized that more independent patients would be more likely to be in stable phase, and vice versa.

*Reliability:* To assess internal consistency, Cronbach's alpha was calculated, with a Cronbach's alpha of ≥ 0.8 indicating good internal consistency.^31^ For the inter-rater reliability, the weighted kappa statistic (*k*) was used to determine the level of agreement between two raters for each domain in the RUG-ADL.

### Ethical considerations

This study was performed according to the Declaration of Helsinki. Ethical approval was obtained from the Human Research Ethical Committee (HREC) at the University of Wollongong (IRB No. 2022/160) and Hunan Cancer Hospital (IRB No. KY2022217). Informed consent was obtained from all patients and clinicians prior to participation by the research team.

## Results

### Cross-cultural adaptation

Minor grammatical and wording discrepancies were identified in the forward translations of the tools, and these were easily resolved through discussion between the two translators. During the cognitive interviews, palliative care clinicians reported that they found both tools easy to use and that no modifications were needed for the AKPS. However, for the RUG-ADL, clinicians emphasized the necessity of clearly explaining there is no score of “2” for the three domains: bed mobility, toilet, and transfer. Additionally, for the item “eating”, clinicians recommended moving the descriptor “patient is unconscious” from the “note” to the instructions for the score of “3” in the PCOC assessment tools manual (The Chinese version of AKPS, RUG-ADL and their introductions are attached as supplementary file I).

### Validation of the AKPS and RUG-ADL

#### Participants characteristics

Twenty two out of 25 clinicians from the palliative care unit and the advanced cancer care unit at a cancer hospital participated in this study. Most were nurses (*n* = 20, 90.9%) and female (*n* = 21, 95.5%), and 40.9% had more than five years of working experience in palliative care. A total of 368 paired assessments were completed for 135 inpatients. The average age of the inpatients was 59.4 ± 11.3 years. The detailed characteristics of the patients are presented in Table 1.

**Table 1:** Patient characteristics (*N* = 135).

Characteristics		*n* (%)
**Age(years)**	< 65	85 (63.0)
	≥ 65	50 (37.0)
**Sex**	Male	85 (63.0)
	Female	50 (37.0)
**Diagnosis**	Lung cancer	53 (39.3)
	Colorectal cancer	22 (16.3)
	Oesophageal gastric cancer	22 (16.3)
	Hepatobiliary pancreatic cancer	14 (10.4)
	Oral cancer	5 (3.7)
	Gynecological cancer	3 (2.2)
	Brain cancer	2 (1.5)
	Prostatic cancer	2 (1.5)
	Other cancers (including for example kidney, bone, pelvic, retroperitoneal, adrenal)	11 (8.1)

#### AKPS

Inter-rater reliability: The greatest discrepancies in AKPS assessments occurred between level 1 (AKPS = 70–100) and level 2 (AKPS = 50 or 60), comprising more than half of the mismatched ratings (61 out of 111 mismatches) (Table 2). There was a substantial strength of agreement between two raters in using the AKPS (Weighted Kappa = 0.63, 95% CI = 0.56–0.70, *P* < 0.001), with a match percentage of 68.9%.

**Table 2:** The inter-rater rating characteristics for AKPS by rater agreement, and the number and proportion of assessments.

	Raters		AKPS (*n* = 357)
	Rater 1	Rater 2	
**Matched ratings**	70–100	70–100	171 (47.9)
	50 or 60	50 or 60	38 (10.6)
	30 or 40	30 or 40	19 (5.3)
	20 or 10	20 or 10	18 (5.0)
	**Total matched**		**246 (68.9)**
**Mismatched ratings**	70–100	50 or 60	35 (9.8)
	50 or 60	70–100	36 (10.1)
	50 or 60	30 or 40	13 (3.6)
	30 or 40	70–100	6 (1.7)
	30 or 40	50 or 60	12 (3.4)
	30 or 40	20 or 10	5 (1.4)
	20 or 10	30 or 40	4 (1.1)
	**Total mismatched**		**111 (31.1)**

AKPS, Australia-modified Karnofsky Performance Status.

Concurrent validity: In alignment with our hypothesis, a high positive correlation was observed between the AKPS score and the BI total score (*r* = 0.77, *P* < 0.01), and a high negative correlation existed between the AKPS sore and the RUG-ADL total score (*r* = −0.82, *P* < 0.001) (Table 3).

**Table 3:** The correlation between RUG-ADL, AKPS and BI.

	RUG-ADL			AKPS (*n* = 330)
	Toileting (*n* = 339)	Transfer (*n* = 342)	Eating (*n* = 340)	
**BI items**				
Feeding	–	–	−0.67∗	–
Toilet use	−0.76∗	–	–	–
Transfer (bed to chair and back)	–	−0.71∗	–	–
**BI total score**	–	–	–	0.77∗
**RUG-ADL total score**	–	–	–	−0.82∗

∗*P* < 0.001. RUG-ADL, Resource Utilization Groups - Activities of Daily Living; AKPS, Australia-modified Karnofsky Performance Status; BI, Barthel index.

Known-groups validity: The results were consistent with our hypothesis that patients with good performance status would be more likely to be in the stable phase rather than in unstable/deteriorating phases. Table 4 presents the percentages of patients at different levels of performance relative to the palliative care phase.

**Table 4:** The ability of the AKPS and the RUG-ADL in differentiating palliative care phases.

	Palliative care phase			*P*
	Stable *n* (%)	Unstable *n* (%)	Deteriorating *n* (%)	
**AKPS (*n* = 352)**				
70–100	197 (56.0)	4 (1.1)	2 (0.6)	
50 or 60	72 (20.5)	6 (1.7)	9 (2.6)	
30 or 40	23 (6.5)	3 (0.9)	14 (4.0)	<0.001
10 or 20	8 (2.3)	4 (1.1)	10 (2.8)	
**RUG-ADL total score (*n* = 352)**				
4–5	200 (56.8)	4 (1.1)	3 (0.9)	
6–13	81 (23.0)	6 (1.7)	14 (4.0)	<0.001
14–17	15 (4.3)	5 (1.4)	14 (4.0)	
18	4 (1.1)	2 (0.6)	4 (1.1)	

Note: Terminal phase excluded due to only one patient assessed as being within the terminal phase. RUG-ADL, Resource Utilization Groups - Activities of Daily Living; AKPS, Australia-modified Karnofsky Performance Status.

#### RUG-ADL

*Internal consistency:* The RUG-ADL demonstrated good internal consistency with a Cronbach's alpha of 0.92 (Supplementary Table 3).

Inter-rater reliability: The number of paired assessments ranged from 361 to 363 for each domain of RUG-ADL. The highest percentage of matched assessments between two raters were observed in the domain of eating (80.9%), followed by bed mobility (72.9%), toileting (69.3%), and transfers (68.6%) (Table 5). A moderate strength of agreement was achieved across all four domains of RUG-ADL (Table 6). The largest proportion of mismatched ratings were observed between independent/supervision only (score “1”) and limited assistance (score “2” for eating, and score “3” for toileting, bed mobility and transfers) within all four RUG-ADL domains (Table 5).

**Table 5:** The inter-rater rating characteristics for RUG-ADL by rater agreement, and the number and proportion of assessments.

	Raters		Bed mobility (*n* = 362)	Toileting (*n* = 362)	Transfers (*n* = 363)	Eating (*n* = 361)
	Rater 1	Rater 2				
**Matched ratings**	1	1	230 (63.5)	190 (52.5)	188 (51.8)	259 (71.7)
	2	2	–	–	–	11 (3.0)
	3	3	13 (3.6)	25 (6.9)	22 (6.1)	22 (6.1)
	4	4	16 (4.4)	27 (7.5)	29 (8.0)	–
	5	5	5 (1.4)	9 (2.5)	10 (2.8)	–
	**Total matched**		**264 (72.9)**	**251 (69.3)**	**249 (68.6)**	**292 (80.9)**
**Mismatched ratings**	1	2	–	–	–	17 (4.7)
	1	3	23 (6.4)	24 (6.6)	21 (5.8)	7 (1.9)
	1	4	7 (1.9)	9 (2.5)	11 (3.0)	–
	1	5	1 (0.3)	2 (0.6)	2 (0.6)	–
	2	1	–	–	–	14 (3.9)
	2	3	–	–	–	7 (1.9)
	3	1	30 (8.3)	29 (8.0)	31 (8.5)	17 (4.7)
	3	2	–	–	–	7 (1.9)
	3	4	8 (2.2)	14 (3.9)	14 (3.9)	–
	3	5	5 (1.4)	4 (1.1)	3 (0.8)	–
	4	1	6 (1.7)	10 (2.8)	12 (3.3)	–
	4	3	6 (1.7)	4 (1.1)	5 (1.4)	–
	4	5	4 (1.1)	5 (1.4)	6 (1.7)	–
	5	1	0 (0.0)	1 (0.3)	0 (0.0)	–
	5	3	2 (0.6)	1 (0.3)	1 (0.3)	–
	5	4	6 (1.7)	8 (2.2)	8 (2.2)	–
	**Total mismatched**		**98 (27.1)**	**111 (30.7)**	**114 (31.4)**	**69 (19.1)**

RUG-ADL, Resource Utilization Groups - Activities of Daily Living.

**Table 6:** The inter-rater agreement for RUG-ADL.

RUG-ADL domains	Weighted Kappa	95% confidence interval	*P*	Strength of agreement	Agreement
**Bed mobility**	0.53	0.45–0.61	< 0.001	Moderate	72.9%
**Toileting**	0.56	0.49–0.64	< 0.001	Moderate	69.3%
**Transfer**	0.56	0.49–0.63	< 0.001	Moderate	68.6%
**Eating**	0.51	0.41–0.62	< 0.001	Moderate	80.9%

RUG-ADL, Resource Utilization Groups - Activities of Daily Living.

Concurrent validity: Aligned with our hypothesis, a high correlation was observed between RUG-ADL toileting scores and BI toilet use scores (*r* = −0.76, *P* < 0.01), and RUG-ADL transfer scores and BI transfer (bed to chair and back) scores (*r* = −0.71, *P* < 0.01). Additionally, a moderate correlation was identified between RUG-ADL eating scores and BI feeding scores (*r* = −0.67, *P* < 0.01) (Table 3).

Known-groups validity: Aligned with our hypothesis, patients exhibiting lower levels of dependency were more likely to be found in the stable phase as opposed to being in phases that were unstable or deteriorating. Table 4 shows the distribution of patients by their level of dependency across the different palliative care phases.

## Discussion

Our study is the first to culturally adapt the AKPS and RUG-ADL for clinical application in the Chinese context, and to examine the psychometric properties of these two tools for application in China. Our study shows that the AKPS demonstrated a substantial level of inter-rater reliability, and moderate levels of inter-rater reliability across all domains of the RUG-ADL. The AKPS and the RUG-ADL was also shown to possess high concurrent validity due to the observed high correlation between the AKPS and the BI total score, the AKPS and the RUG-ADL total score, as well as the corresponding items in the RUG-ADL and the BI (expect for the “eating” item). In terms of the known-groups validity, the tools could be used by a range of health care professionals to effectively predict patients in a palliative care stable phase or unstable/deteriorating phase. Additionally, the RUG-ADL demonstrated good internal consistency. Also, only minor modifications to the instruction manual for the RUG-ADL to facilitate its application by Chinese clinicians, with no changes to the AKPS, suggesting many synergies between the Australian and Chinese clinical practice context.

Most mismatches in AKPS ratings occurred between level 1 (AKPS = 70–100) and level 2 (AKPS = 50 or 60), particularly at the threshold determining whether patients require assistance. Similarly, for the RUG-ADL, the most significant discordance was also observed at the critical juncture between independent/supervision only (score “1”) and limited assistance (score “2” for eating, and score “3” for toileting, bed mobility and transfer) across all four RUG-ADL domains. These findings may be influenced by both cultural and clinical factors. In some cultures, there may be a reluctance to admit the need for assistance, as self-reliance is highly valued. Clinically, variations in how clinicians interpret and apply AKPS and RUG-ADL thresholds can also contribute to these mismatches. Such discrepancies highlight the importance of standardized assessment protocols and ongoing training to reduce subjective variability. By recognizing the impact of cultural and clinical factors on person-centered assessments in palliative care, clinicians should incorporate more observational assessments and engage in open communication with patients and families/carers to understand their cultural context and personal preference. This approach ensures that the assessments accurately reflect the patient's performance status, allowing the care plans are tailored to meet individual needs.

In the PCOC model, Palliative Care Phase serves as a tool to identify the clinical acuity and urgency of care needs of patients and/or their families/caregivers.^9^^,^^12^ Our study found that both the AKPS and the RUG-ADL could predict patients in stable and unstable/deteriorating phases, indicating that these assessments can guide clinicians in providing an appropriate and timely clinical response to patients and/or their families/caregivers in a Chinese context. Furthermore, our findings are supported by a previous study, which demonstrated that a lower level of performance status (a lower AKPS score) and higher level of dependency (a higher RUG-ADL mean score) were associated with increased physical/functional needs among palliative care patients.^28^ This correlation reinforces the value of incorporating systematic assessments like the AKPS and RUG-ADL into routine palliative care practices to ensure high-quality care.

The observed moderate correlation between the RUG-ADL “eating” item and the BI “feeding” item, contrary to our initial hypothesis of a high correlation, may be attributed to the thresholds for assistance being interpreted differently by raters using each tool. To minimize subjective interpretation, a more detailed guideline and or enhanced education on the application of the RUG-ADL is recommended. For national application, more detailed guidelines to support clinical use might be more cost efficient.

Although the Chinese versions of the KPS and BI are the most widely used tools to assess patient's performance status and daily capacity in palliative settings in China,^32^, ^33^, ^34^ they have certain limitations when applied in this context. A previous study has highlighted that the language of KPS may not be suitable or flexible enough to meet the diverse needs and settings of palliative care across different clinical environments.^4^ To address this limitation, the AKPS was developed by integrating the KPS with the TKPS and modifying its language to be less directive about the expected location of care, and is more applicable across a wider range of palliative care settings.^4^ Additionally, the AKPS has been shown to be more predictive of survival at the lower end of the scale,^4^ which is significant given these lower levels were assessed more accurately in our study. This accuracy at lower levels may make the AKPS particularly valuable for palliative care populations, where precise assessment of decline is crucial for providing timely end-of-life care.

The BI is useful for measuring physical disability and focuses on a broader range of ADLs, it may not capture the specific functional dependency of palliative care patients, while the RUG-ADL focuses on key areas of functional dependency relevant to palliative care patients – bed mobility, toileting, transfers, and eating. Another significant strength of the RUG-ADL is that it includes detailed instructions for resource allocation, enabling effectively allocation of staff time and other resources based on each patient's specific needs.

Due to their applicability, specificity and predictive accuracy, The AKPS and RUG-ADL are integral components of the PCOC model used in Australia. Adapting and validating these tools in China could similarly enhance palliative care practices by providing more accurate assessments, improving care planning, and facilitating effective resource allocation for palliative care patients.

### Limitations

This study has several limitations. First, the sample was confined to palliative care clinicians and cancer patients in a single hospital, which may limit the generalizability of the findings. Future research should aim to include a wider variety of settings and patient diagnoses within the Chinese context. Additionally, although previous evidence suggested that the AKPS may serve as a predictive marker for survival,^4^ we did not assess its predictive validity in this study. Future studies should focus on conducting longitudinal study to rigorously test its capacity to predict survival time for patients. Third, the uneven distribution of patients across the palliative care phases, particularly the smaller number of patients in the terminal phase, may limit the generalizability of our findings to these groups. However, the data from these phases still provide valuable insights into how the tools differentiate between phases.

## Conclusions

The Chinese version of AKPS exhibits substantial inter-rater reliability, and good concurrent validity, and the RUG-ADL shows moderate inter-rater reliability, good internal consistency, and good concurrent validity. Additionally, both tools demonstrate ability in differentiating patient palliative care phases. However, it is recommended that the use of these tools be supplemented with more observational assessments and enhanced communication between clinicians and patients/caregivers.

## CRediT authorship contribution statement

**Yunyun Dai**: Conceptualization, Methodology, Data curation, Formal analysis, Writing. **Jinfeng Ding**: Conceptualization, Methodology, Writing – Revised draft preparation, Writing – Review and editing. **Barbara A Daveson**: Conceptualization, Methodology, Writing – Revised draft preparation, Writing – Review and editing. **Yongyi Chen**: Conceptualization, Methodology, Writing – Review and editing. **Alanna Connolly**: Formal analysis, Writing – revised draft preparation. **Claire E Johnson**: Conceptualization, Methodology, Writing – Revised draft preparation, Writing – Review and editing, and Supervision. All authors had full access to all the data in the study, and the corresponding authors had final responsibility for the decision to submit for publication. The corresponding authors attest that all listed authors meet authorship criteria and that no others meeting the criteria have been omitted.

## Ethics statement

The study was approved by the Human Research Ethical Committee (HREC) at the University of Wollongong (IRB No. 2022/160) and Hunan Cancer Hospital (IRB No. KY2022217). All participants provided written informed consent.

## Funding

This work was supported by Guilin Science and Technology Bureau (Grant No. 20220139-6-4) and Hunan Provincial Natural Science Foundation Youth Program (Grant No. 2023JJ40791). PCOC is funded by the Australian Government Department of Health and Aged Care. The funders had no role in considering the study design or in the collection, analysis, interpretation of data, writing of the report, or decision to submit the article for publication.

## Data availability statement

The data that supports the findings of this study are available on request from the corresponding author, Yunyun Dai, upon reasonable request.

## Declaration of generative AI and AI-assisted technologies in the writing process

No AI tools/services were used during the preparation of this work.

## Declaration of competing interest

The authors declare no conflict of interest. Professor Yongyi Chen, the corresponding author, serves on the editorial board of the *Asia-Pacific Journal of Oncology Nursing*. The article underwent standard review procedures of the journal, with peer review conducted independently of Professor Chen and their research groups.
