# Supplementary material for: Validating performance status and activities of daily living assessment tools for Chinese palliative care in a cancer setting: A cross-cultural psychometric study
Source: Asia Pac J Oncol Nurs. 2024 Oct 29;11(12):100613. doi: 10.1016/j.apjon.2024.100613 (PMC11617370; doi:10.1016/j.apjon.2024.100613)
Supplement: Multimedia component 1 [file mmc1.docx]

**Supplementary table 1. Participants characteristics for cognitive interview**

| **Participants characteristics** | | **N** |
| --- | --- | --- |
| **Clinicians (N=6)** | | |
| Age (Mean, Range) | | 32.0 (29-35) |
| Gender | Male | 1 |
|  | Female | 5 |
| Occupation | Doctor | 3 |
|  | Nurse | 3 |
| Work experience in palliative care (Year) | <5 | 2 |
|  | ≥5 | 4 |
| PCOC training | Yes | 6 |
|  | No | 0 |

**Supplementary table 2. Questions for the cognitive interviews**

| **AKPS and RUG-ADL** |
| --- |
| 1. Do you find the definition/instruction for AKPS/RUG-ADL easy to understand? |
| 1. Is it difficult for you to assess patients by using AKPS/RUG-ADL? |
| 1. Are there some parts of AKPS/RUG-ADL that need to be modified? |
| 1. Do you think AKPS/RUG-ADL is useful in guiding your clinical practice? |

**Supplementary table 3. Internal consistency of RUG-ADL (N=No. Of assessments)**

| **RUG-ADL Items** | **Score N (%)** | | | | | **Score, Mean (SD)** | **Cronbach alpha** |
| --- | --- | --- | --- | --- | --- | --- | --- |
|  | **1** | **2** | **3** | **4** | **5** |  |  |
| Bed mobility (N=362) | 267 (73.8) | - | 43 (11.9) | 37 (10.2) | 15 (4.1) | 1.71±1.23 | 0.92 |
| Toileting (N=362) | 231 (63.8) | - | 54 (14.9) | 57 (15.7) | 20 (5.5) | 2.00±1.39 |  |
| Transfer (N=362) | 231 (63.8) | - | 49 (13.5) | 61 (16.9) | 21 (5.8) | 2.01±1.41 |  |
| Eating (N=367) | 291 (80.4) | 35 (9.7) | 36 (9.9) | - | - | 1.29±0.64 |  |

**The supplementary file I**

| **PCOC评估量表 （PCOC assessment tools）** | | |
| --- | --- | --- |
| **医护人员评分** Clinician rated score | **澳大利亚改良版-卡诺夫斯基功能状态量表（AKPS, 0-100）**The Australia-modified Karnofsky Performance Status (AKPS, 0-100)  等于或少于50分考虑多学科团队进行评估 Consider MDT review at score of 50 or below | |
|  | AKPS |  |
|  | **资源利用分群-日常生活能力量表(RUG-ADL)** The Resource Utilization Groups - Activities of Daily Living (RUG-ADL)  4 - 5 = 持续监测Monitor  6 - 10 = 一人协助 assist x 1  10+ = 提供帮助，考虑使用器械和人员帮助，有跌倒风险，转诊assist x 1, consider equipment, staff requirements, falls risk, referral  15+ = 同上，有发生压疮的风险，考虑照顾人压力，多学科团队评估 as above, pressure area risk, consider carer burden and MDT review  18 = 同上，全面照护协助as above, full care assistance x 2 | |
|  | 床上活动能力 Bed mobility |  |
|  | 如厕能力 Toileting |  |
|  | 转移能力 Transfers |  |
|  | 进食能力 Eating |  |
|  | 总分Total RUG-ADL (4-18): |  |
|  | **安宁疗护阶段** Palliative Care Phase  1.稳定阶段=监测 Stable = Monitor  2.不稳定阶段=需紧急处理 Unstable = Urgent action required  3.恶化阶段=对照护计划进行审查，评估照护计划是否有效 Deteriorating = Review plan of care  4.终末阶段=提供临终照护服务 Terminal = Provide EOL care | |
|  | 安宁疗护阶段Palliative Care Phase |  |

**PCOC评估量表使用说明 (PCOC assessment tools manual)**

| **澳大利亚版-改良卡若夫斯基功能状态量表（AKPS）** | |
| --- | --- |
| **医护人员对患者在过去24小时内有关于工作、活动和自我照护能力表现的评估**Clinician rated assessment of performance relating to work, activity and self-care over a 24hr period  100分 正常，没有疾病症状或征象Normal, no complaints or evidence of disease  90分 能够进行正常活动，有轻微的疾病症状或征象Able to carry on normal activity, minor signs or symptoms of disease  80分 勉强正常活动，有一些疾病的症状或征象Normal activity with effort, some signs or symptoms of disease  70分 生活自理，但无法维持正常活动或主动工作Care for self, unable to carry on normal activity or to do active work  60分 偶尔的协助，多数情况下生活自理Occasional assistance but is able to care for most needs  50分 需要很多协助和医疗照护Requires considerable assistance and frequent medical care  40分 卧床时间超过50%In bed more that 50% of the time  30分 几乎完全卧床Almost completely bedfast  20分 完全卧床，需要医护人员和/或家属照护Totally bedfast & requiring nursing care by professionals and/or family  10分 昏迷或几乎叫不醒Comatose or barely rousable  0分 死亡 Death | |
| **资源利用小组-日常生活能力量表（RUG-ADL）** | |
| **医护人员对患者在过去24小时内独立性的评估**Clinician rated assessment of dependency over 24hr period | |
| **床上活动、如厕和移动 (评分无“2分”选项)** For Bed Mobility, Toileting & Transfers (there is no score of “2”)  1分 独立或只需要监督Independent or supervision only  3分 需一定程度的协助Limited physical assistance  4分 一人协助Other than two person physical assist  5分 两人或多人协助Two or more person physical assist | **进食**For Eating  1分 独立或只需监督Independent or supervision only  2分 需一定程度的协助Limited assistance  3分 需很大程度的协助/完全依赖/管道喂养/昏迷 Extensive assistance / total dependence / tube fed/unconscious. |
| **安宁疗护阶段(Palliative Care Phase)** | |
| **稳定阶段：**目前所采取的照护措施能有效管控患者的症状和问题。继续监测患者需求、审查照护计划、预期患者可能的变化并及时应对。**Stable:** Symptoms and problems are adequately controlled by established management. Monitor, review, anticipate & respond.  **不稳定阶段**：患者出现新的问题，或患者现存的问题、或家属/照顾者问题的迅速恶化，需要马上更改照护计划或进行紧急治疗。需采取紧急应对措施。**Unstable:** An urgent change in the plan of care or emergency treatment is required due to development of a new problem &/or a rapid increase in the severity of existing problems &/or family/carer problems. Urgent response required.  **恶化阶段：**患者的功能状态不断的下降，和/或现有症状不断的恶化，和/或出现了预期范围内的新问题，和/或家属/照顾者出现了预期范围内的新问题。照护计划正在尽力解决这些预期的问题但仍需对患者进行定期评估。审查和更改照护计划。**Deteriorating:** The plan of care is addressing anticipated needs but requires periodic review due to gradual functional decline &/or worsening of existing symptoms &/or the development of new but expected problems &/or family/carer problems. Review & change care plan.  **终末阶段：**患者可能在近日内死亡。提供临终照护。**Terminal:** Death likely in a matter of days. Monitor, review & respond. | |
