# Supplementary material for: Validating performance status and activities of daily living assessment tools for Chinese palliative care in a cancer setting: A cross-cultural psychometric study
Source: Asia Pac J Oncol Nurs. 2024 Oct 29;11(12):100613. doi: 10.1016/j.apjon.2024.100613 (PMC11617370; doi:10.1016/j.apjon.2024.100613)
Supplement: Multimedia component 2 [file mmc2.docx]

**
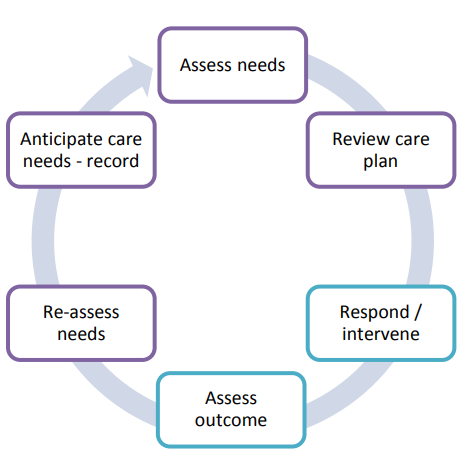
**

**Supplementary figure 1 PCOC routine clinical assessment and response framework**

**
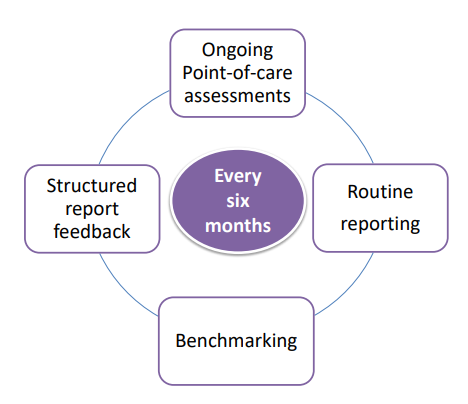
**

**Supplementary figure 2 PCOC cycle**
